# Impact of expression systems on the transcriptome of *Bacillus subtilis*: insights for enhanced production of glutaminase PrgA

**DOI:** 10.1128/aem.01374-25

**Published:** 2025-09-04

**Authors:** Mariah B. M. J. Kes, Biwen Wang, Joen Luirink, Leendert W. Hamoen

**Affiliations:** 1 Molecular Microbiology, Amsterdam Institute of Molecular and Life Sciences, Vrije Universiteit Amsterdam 1190, Amsterdam, The Netherlands; 2 Bacterial Cell Biology, Swammerdam Institute for Life Sciences, University of Amsterdam, Amsterdam, The Netherlands; 3 Institute for Life Science and Technology, Research Centre Biobased Economy, Hanze University of Applied Sciences 3645, Groningen, The Netherlands; University of Milano-Bicocca, Milan, Italy

**Keywords:** CtsR, nanobody, protein glutaminase, xylanase, RNA sequencing, protein secretion, *Bacillus subtilis*

## Abstract

The gram-positive bacterium *Bacillus subtilis* is widely used for enzyme production, especially due to its superior protein secretion capacity. In this study, we have investigated how efficient transcriptome analysis can identify general and protein-specific secretion stress. For this, we constructed *B. subtilis* strains overproducing different commercially relevant proteins, including a GFP-specific camelid nanobody (GFPnb), the xylanase XynA and the protein glutaminase PrgA, and expressed these proteins either from the strong constitutive P*amyQ* promoter or from the xylose-inducible P*xyl* promoter. The use of the inducible P*xyl* promoter influenced the expression of many more genes than when the constitutive P*amyQ* promoter was used to express the different proteins. Presumably, the constitutive expression gives cells time to adapt to the imposed secretion stress. These results underscore the importance of performing transcriptome analyses under conditions that most closely mimic industrial production settings. Finally, we tested whether the transcriptome data could provide clues to improve the production of PrgA. The secretion stress response upon PrgA expression appeared similar to that observed upon alpha-amylase overproduction. However, mutants that would normally improve alpha-amylase production did not result in better PrgA yields. Overexpression of PrgA also results in the downregulation of several Clp protein chaperones. Interestingly, induction of these genes, by inactivating their transcriptional repressor CtsR, strongly improved PrgA production. This analysis highlights the limitations, as well as the potential use of transcriptome comparisons to uncover production bottlenecks.

**IMPORTANCE:**

The bacterium *Bacillus subtilis* is widely used in bioindustry to produce various proteins. However, not all proteins are efficiently produced. In this study, we examined how *B. subtilis* responds to the production of three different industrially relevant proteins. By analyzing gene activity using RNA sequencing, we identified factors that limit protein production and successfully improved the yield of one protein by 2.7-fold. However, our results also show that the choice of promoter used significantly affects transcriptomic regulation. This highlights the importance of testing production conditions that closely resemble relevant industrial settings to identify the best strategies for improving industrial protein yields.

## INTRODUCTION

*Bacillus subtilis* is a gram-positive bacterium widely used for the production of bulk enzymes due to its superior protein secretion capacity, which simplifies and economizes enzyme isolation (1). Additionally, *B. subtilis* is amenable to genetic manipulation and holds the “generally recognized as safe” (GRAS) status, a critical requirement for applications in the food industry. Despite these advantages, high production yields are currently limited to a select number of enzymes. A first step toward improved yields involves removal of proteases and optimizing the promoter, signal peptide, and fermentation conditions (2–4). However, even then, most heterologous proteins are expressed at levels below the maximum production capacity of *B. subtilis*, which can reach 20–25 g/L (5). The combined impact of expression system and target protein, and which bottlenecks limit high production yields is in most cases unknown.

Genome-wide transcriptome analysis provides a powerful and unbiased method to monitor cellular stress responses associated with protein overexpression, offering insights into previously unrecognized bottlenecks. Despite its potential, only a limited number of studies have employed this approach in the context of secreted protein production (6–10). Notably, it has been instrumental in enhancing the production of the xylanase XynA, where transcriptome profiling identified the downregulation of *clp* protein chaperon genes as a bottleneck (7). Another study compared transcriptomic responses in cells overproducing secreted proteins, lipoproteins, and membrane proteins (6). Such a comparison is particularly useful, because it can indicate specific and general secretion bottlenecks. However, this analysis was conducted during the logarithmic growth phase. Given that *B. subtilis* secretes most of its extracellular proteins during the stationary phase, driven by the demand for nutrient-scavenging enzymes such as proteases and amylases (11–13), a stationary-phase perspective is essential for uncovering relevant stress responses. Therefore, in this study, we performed transcriptomic profiling during stationary phase while expressing three different commercially relevant proteins. Our aim was to identify both general cellular stresses associated with high-level secretion and protein-specific stresses, ultimately to inform targeted strategies for improving protein production yields, and to assess the usefulness of transcriptome analysis for the identification and mitigation of relevant secretion bottlenecks.

To ensure clear protein-dependent transcriptional stress signatures, we compared the transcriptomes of *B. subtilis* producing three proteins with distinct properties based on size and isoelectric point (pI): the small 13.7 kDa GFP-specific camelid nanobody (GPFnb, pI = 6.44) (14), the 20.4 kDa xylanase XynA from *B. subtilis* (pI = 9.05) (15), or the 32.7 kDa protein glutaminase PrgA from *Chryseobacterium proteolyticum* (pI = 8.41) (16). Notably, the production of PrgA by *B. subtilis* is inefficient, leading to particularly low yields (17, 18). These proteins were expressed using the strong constitutive P*amyQ* promoter (19). However, continuous high-level production may impose fitness costs on the host, due to the metabolic burden and potential toxicity of the overexpressed proteins, which can lead to reduced yields or the emergence of secondary mutations (20). Therefore, we repeated the transcriptome analyses under the same conditions, but this time using the strong xylose-inducible promoter P*xyl* (21). This had a profound effect on the transcriptome, suggesting that continuous overproduction of secreted proteins leads to cellular adaptation. Nevertheless, certain protein-specific stress responses remained fairly constant, and using this information, we tested several mutant background strains to examine whether PrgA levels could be improved. Importantly, we found that inactivation of the transcriptional repressor CtsR resulted in a 2.7-fold increase in PrgA levels.

## RESULTS

### Transcriptome profiles using constitutive expression conditions

For the overexpression of proteins, we used *B. subtilis* host strain BWB143, which lacks *spoIIE*, rendering it incapable of sporulation. It also lacks the primary secreted feeding proteases NprE and AprE (22), which are typically secreted during the stationary phase and known to significantly reduce the yield of many overexpressed heterologous proteins in the medium (23–25). The genes encoding GFPnb, XynA, or PrgA were cloned under the control of the strong constitutive promoter P*amyQ* (19) in a multicopy plasmid derived from pUB110 that contains a kanamycin resistance marker for selection (26). The empty expression vector served as a non-expression control. To ensure good secretion of GFPnb and PrgA in *B. subtilis*, these constructs contained the YoaW signal peptide from *B. subtilis*, which works well for the secretion of heterologous proteins in *Bacillus* species (27). In contrast, XynA, a native *B. subtilis* enzyme, retained its endogenous signal peptide, which gives excellent yields (7). Finally, a 6x-histidine tag was fused to the C-terminus of GFPnb and PrgA to enable detection of the proteins in the medium by western blot analysis.

 We first examined the production of the different proteins in the medium to determine the RNA sampling time point. Strains were grown in LB medium, and at different time points in the stationary phase, medium samples were taken for SDS-PAGE analysis. The production of these proteins did not affect the growth rate of the host strain (Fig. S1, left panel). After 5 h of growth, when the cultures were in the stationary phase, GFPnb and XynA appeared noticeably produced, as indicated by the appearance of distinct protein bands of the expected size in SDS-PAGE (Fig. S2A, lanes 4 and 6). PrgA was only weakly visible, consistent with previous reports that highlight the challenges of its overproduction in *B. subtilis* (Fig. S2A, lane 8) (17, 18). Since GFPnb and XynA are clearly produced after 5 h of growth, this time point was chosen for harvesting cells for RNA isolation. The experiments were performed two times to obtain biological replicates. All transcriptome data generated in this study are provided in Table S1.

**Fig 1:**
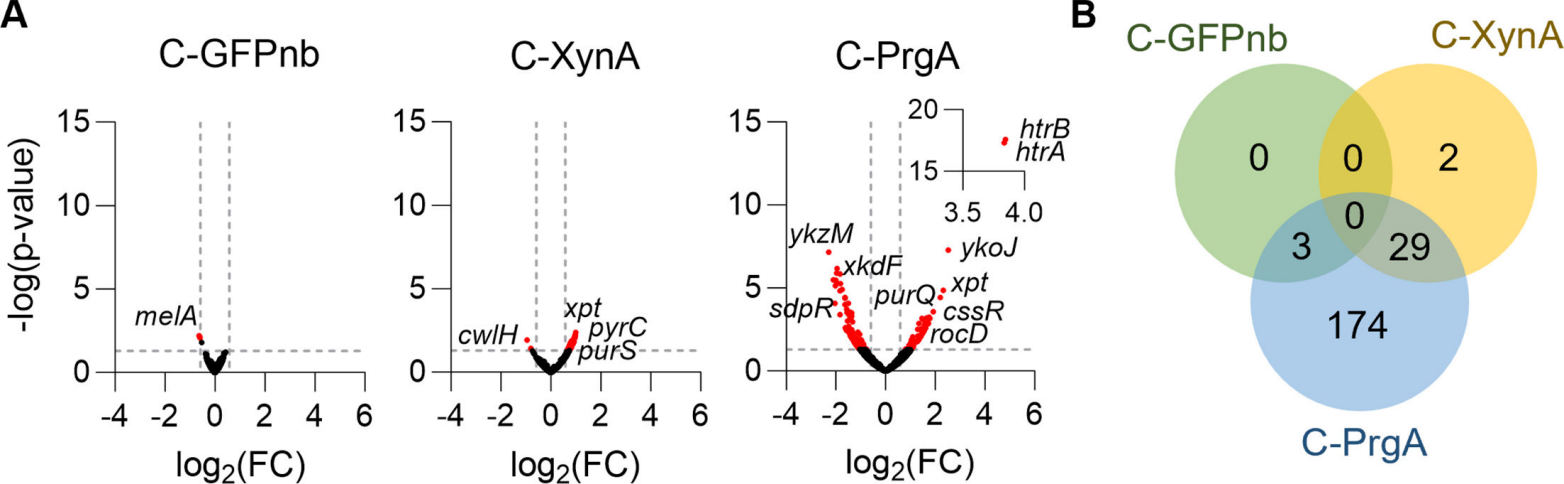
Transcriptome analysis of constitutively overproducing GFPnb, XynA, and PrgA strains. (**A**) Volcano plots of fold change (FC) versus *P* values. Dashed lines represent FC > 1.5 (log2 > 0.6) and *P* values < 0.05 cut-offs. (**B**) Venn diagram of the genes that show significant expression differences in the three strains. Data for the *gfpnb*, *xynA*, and *prgA* genes are not shown.

To compare gene expression differences between the strains, the transcriptome data were visualized as volcano plots (Fig. 1A). Notably, the production of PrgA had the most pronounced impact on the expression profiles. To focus on the genes that showed relevant expression differences, we used a fold change > 1.5 (log2 > 0.6) and a *P* value < 0.05 as threshold levels. This resulted in 206 genes that were significantly up- or downregulated. In contrast, only 3 genes showed significant expression changes in the GFPnb overexpressing strain and 31 genes in the XynA overexpressing strain. The Venn diagram in Fig. 1B shows that the expression of these genes is also significantly affected in the PrgA-producing strain.

Table 1 lists the 10 most strongly up- and downregulated genetic loci in response to production of PrgA and compares these with the GFPnb- and XynA-producing strains. Among the most prominent effects of PrgA overexpression is the induction of the extracellular membrane-attached quality control proteases *htrA* and *htrB*, which are also moderately upregulated in response to constitutive XynA production. The upregulation of these proteins is a well-known response to the overproduction of secreted proteins (28, 29). In contrast, GFPnb production does not significantly upregulate these genes. *ykoJ*, whose function is unknown, is also strongly upregulated in PrgA producing cells, but not in GFPnb- or XynA-producing cells. Most genes in Table 1 are involved in metabolic processes or part of prophage elements, and they are unlikely to represent bottlenecks for protein secretion. Finally, *cssR* encodes the two-component response regulator that activates transcription of *htrAB* (28, 29), and the *bdbB* gene encodes an extracellular membrane-bound thiol-disulfide oxidoreductase involved in oxidative folding of proteins (30, 31). The latter is likely activated because of the four disulfide bonds that are present in mature PrgA (32).

**TABLE 1:** Top 10 up- and downregulated genetic loci in response to constitutive overexpression of *prgA*^*a*^

Gene(s)	PrgA	GFPnb	XynA	Function
*htrB*	3.8	−0.0	0.7	Membrane-anchored protein quality control protease
*htrA*	3.8	0.1	0.7	Membrane-anchored protein quality control protease
*ykoJ*	2.5	0.0	−0.0	Unknown
*xpt-pbuX*	2.2/2.3	0.2/0.3	0.8/1.0	Purine salvage and acquisition
*purEKBCSQLFMND*	1.5/1.9	0.1/0.4	0.5/1.0	Purine biosynthesis
*pyrC*	1.6	0.1	0.8	Pyrimidine biosynthesis
*rocD*	1.6	0.1	0.6	Arginine, ornithine, and citrulline utilization
*bdbB*	1.6	0.2	0.9	Oxidative folding of proteins
*pbpX*	1.5	0.1	0.6	Endopeptidase (penicillin-binding protein X)
*cssR*	1.5	−0.0	0.1	Two-component response regulator that activates transcription of *htrAB*
*ykzM*	−2.3	0.0	−0.1	Unknown PBSX prophage protein
*xtmB-xkdEFJKMNPQRSTU-xkzA-xkdVWX-xepA-xhlAB-xlyA*	−2.1/−1.4	−0.0/0.1	−0.2/0.1	PBSX prophage
*sdpR*	−2.0/−1.8	0.0/0.0	−0.4/−0.3	Protection against SdpC toxin
*ykzL*	−2.0	−0.0	−0.1	Unknown PBSX prophage protein
*hutHUIGM*	−1.6 / −1.6	0.0/0.0	−0.3/−0.2	Histidine utilization
*araABDLMNP*	−1.6/−1.4	−0.0 / −0.0	−0.1/−0.0	Arabinose utilization
*iolT*	−1.6	0.0	−0.1	Myo-inositol uptake
*yxiB*	−1.5	−0.0	−0.3	Unknown
*licB*	−1.4	−0.1	−0.3	Lichenan uptake and phosphorylation, control of LicR activity
*iolB*	−1.4	0.0	−0.1	Myo-inositol catabolism

^*a*^ Fold changes (log2) of the most affected genes in the PrgA-producing strain. Genes in an operon are considered as a single genetic locus, whereby the minimum and maximum FC values are indicated.

### Transcriptome profiles under induced expression conditions

To investigate whether induced overexpression of GFPnb, XynA, and PrgA triggers a different transcriptome response compared to constitutive overexpression, we used the same *B. subtilis* strain and plasmid system but replaced the constitutive P*amyQ* promoter with the strong xylose-inducible P*xyl* promoter (21). After initial dilution, cells were grown for 3 h when expression was induced by the addition of 1% xylose to the medium. This induction had no observable impact on growth (Fig. S1, right panel). We aimed to achieve expression levels on the same order of magnitude as those observed after 5 h of constitutive production and found that this was reached at approximately 2 h post-induction, as judged by SDS-PAGE (Fig. S2A**,** lanes 3, 5, and 7). This time point was chosen for the isolation of RNA. Of note, over time, the GFPnb protein band migrated further in the gel, indicative of proteolytic processing (Fig. S2B). In the constitutive GFPnb-expressing culture, only the processed version is observed on gel (Fig. S2A).

**Fig 2:**
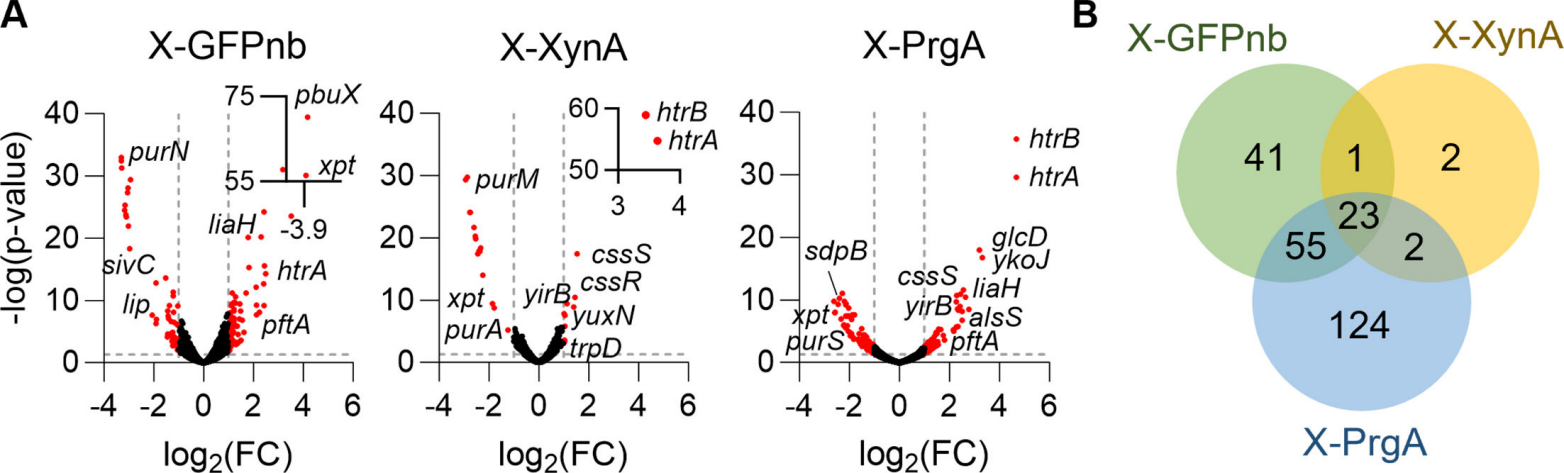
Transcriptome analysis of xylose-induced overproducing GFPnb, XynA, and PrgA strains. (**A**) Volcano plots of fold change (FC) versus *P* values. Dashed lines represent FC > 2 (log2 > 1) and *P* values < 0.05 cut-offs. (**B**) Venn diagram of the genes that show significant expression differences in the three strains. Data for the *gfpnb*, *xynA*, and *prgA* genes are not shown.

Induced overexpression resulted in extensive transcriptional changes (Fig. 2A). The expression of 121 genes changed more than twofold (*P* value < 0.05) when expression of the camelid nanobody was induced, compared to 28 and 204 genes after induction of XynA and PrgA, respectively. The Venn diagram in Fig. 2B shows that there is a substantial overlap between the differentially expressed genes in the three conditions.

Table 2 lists the 10 most strongly up- and downregulated genetic loci for each strain. The strongest response in all conditions is the induction of the *htrAB* secretion stress locus. *liaIH* expression has been correlated to cell envelope targeting antibiotics and oxidative stress (33), and *yirB* is involved in stabilizing Spx in response to cell wall stress (34, 35). The transcriptional regulator Spx regulates many genes, including those important for the prevention of protein aggregation during heat stress and protection against oxidative stress (36, 37). *nfrA*, encoding a nitro/flavin reductase, is under control of Spx (34). Most genes in Table 2 show a similar pattern of regulation across the different expression conditions, indicating that the most prominent transcriptional changes are related to a general stress response triggered by the overexpression of secreted proteins.

**TABLE 2:** Top 10 up- and downregulated genetic loci in response to the induced secreted protein overexpression^*a*^

Gene(s)	GFPnb	XynA	PrgA	Description
*htrA*	2.4	3.6	4.7	Membrane-anchored protein quality control protease
*htrB*	2.4	3.5	4.7	Membrane-anchored protein quality control protease
*dctP*	3.5	0.4	1.5	Uptake of succinate, fumarate, malate, and oxaloacetate
*ykoJ*	0.2	1.0	3.3	Unknown
*glcDF*	1.5/2.4	0.4/0.7	2.6/3.2	Probable glycolate oxidase
*maeN*	2.1	0.8	2.8	Malate uptake
*liaIH*	2.1/2.3	0.6/0.7	2.5/2.6	Resistance against oxidative stress and cell wall antibiotics
*yyzE-bglA*	2.4/2.5	1.0/1.0	1.0/1.1	Beta-glucoside utilization
*alsS*	0.4	0.1	2.5	Overflow metabolism
*cssRS*	1.0/1.1	1.5/1.5	2.4/2.4	Two-component response regulator that activates transcription of *htrAB*
*pftAB*	2.1/2.2	0.9/1.0	2.2/2.3	Uptake of pyruvate
*yirB*	1.0	1.4	2.3	Stabilization of Spx in response to cell wall stress
*treP*	1.8	0.4	2.3	Trehalose uptake and phosphorylation
*nagP*	1.8	0.6	0.4	N-acetylglucosamine uptake and phosphorylation
*nfrA*	1.3	1.1	1.1	Spx-dependent FMN-containing NADPH-linked nitro/flavin reductase, stress protein
*yuxN*	0.9	1.0	1.2	Control of Spx stability, represses *yirB*
*yhzC*	1.1	1.1	1.1	Unknown
*ylbP*	1.0	1.1	0.4	Unknown
*trpD*	0.0	1.0	0.1	Biosynthesis of tryptophan
*xpt-pbuX*	−3.9/−3.9	−1.9/−1.8	−2.6/−2.6	Purine salvage and acquisition
*purEKBCSQL* *FMNHD*	−3.3/−2.9	−2.9/−2.2	−2.3/−1.8	Purine biosynthesis
*sivC*	−1.9	−1.0	−2.6	Inhibitor of entry into sporulation
*sdpABC*	−1.4/−0.9	−0.6/−0.3	−2.5/−2.0	Killing of non-sporulating sister cells
*ppsABCDE*	−1.1/−0.2	−0.7/−0.2	−2.3/−2.0	Production of the antibacterial compound plipastatin
*mntABCD*	−1.4/−1.3	−0.5/−0.4	−2.2/−2.1	Manganese uptake
*malA-glvR-malP*	−2.1/−1.9	−0.9/−0.8	−2.0/−1.5	Maltose utilization
*pksJL*	−1.0/−0.7	−0.6/−0.4	−1.9/−1.8	Polyketide synthesis
*yscB*	−1.2	−0.6	−1.9	Unknown
*ctaC*	−0.5	−0.1	−1.7	Cytochrome-c oxidase (subunit II), respiration
*yecA*	−1.5	−0.9	−1.0	Putative transporter
*pbuG*	−1.5	−0.9	−0.7	Hypoxanthine and guanine uptake
*lip*	−1.4	−0.9	−1.1	Lipid degradation
*purA*	−1.3	−1.2	−0.5	Purine biosynthesis
*melECA*	−1.3/−1.2	−1.0/−0.9	−0.6/−0.6	Melibiose utilization
*steT*	−0.2	−1.0	−0.3	Exchange of serine and threonine

^*a*^ Fold changes (log2) of the most affected genes in each strain are listed. Genes in an operon are considered as a single genetic locus, whereby the minimum and maximum FC values are indicated.

### Comparing expression conditions through regulon analysis

**Fig 3:**
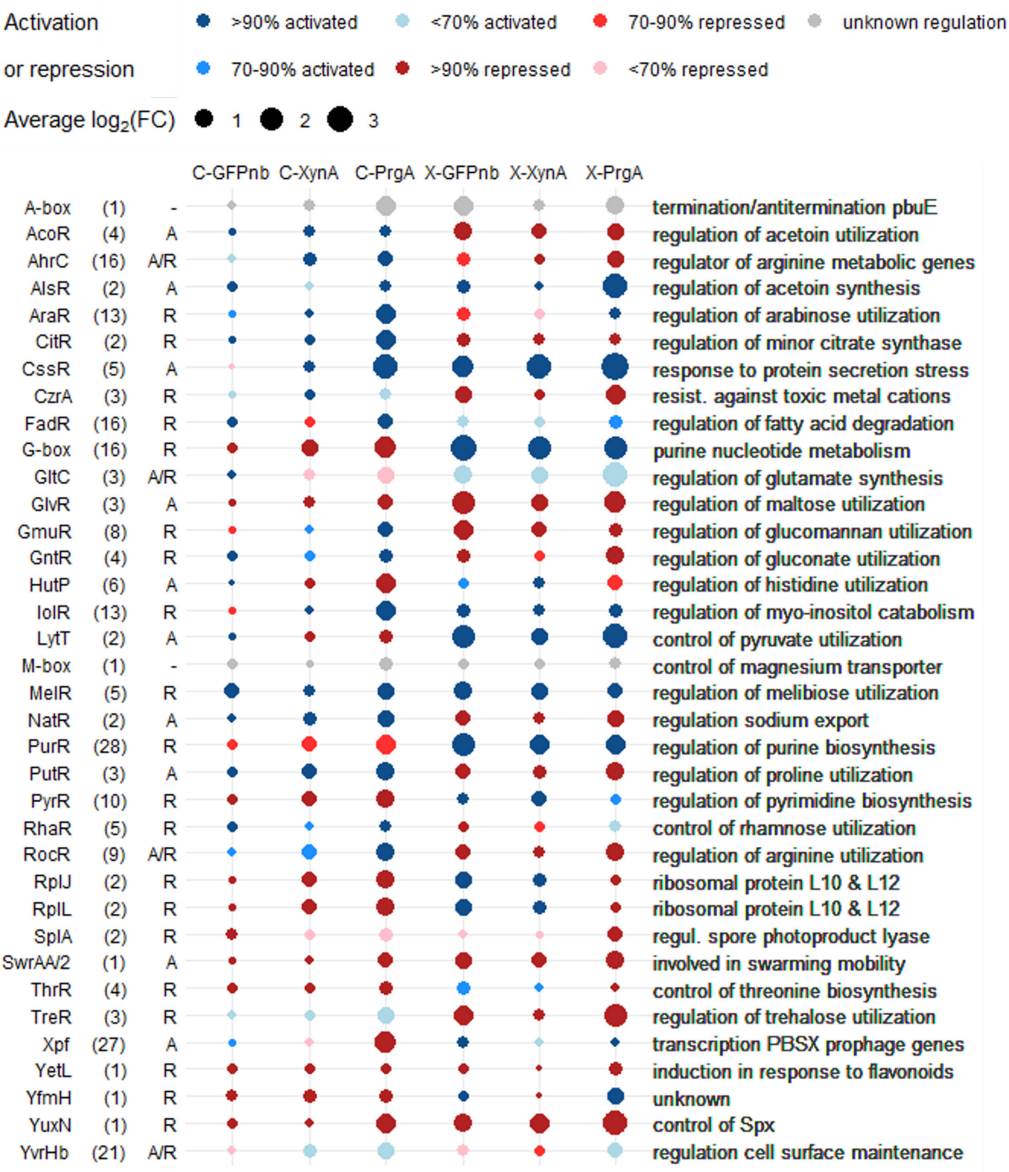
Comparison of regulon activities of overproduced proteins using two distinct promoter systems. The transcriptome data were analyzed with GINtool, and the regulon activities of the top five repressed and activated regulons of each condition are shown. Bubble size represents the absolute average fold change. The total size of the regulon is shown in parentheses behind the regulon name. Color intensities represent the number of genes in a regulon that show the same activity, either by an activated (blue) or repressed (red) regulator. A/R/- indicates whether a regulator is an activator, repressor, or has an unknown activity, respectively. Thus, a red regulon controlled by a repressor (R) means that the genes of this regulon are actually activated. Gray represents unknown regulation directionality.

The large differences between constitutive and induced expression raises the question of whether we can distinguish common regulation responses for each protein. To facilitate this analysis, we focused on signal transduction pathways by analyzing the activity of regulons. *B. subtilis* is one of the most extensively studied bacterial species, and so far, over 200 regulons have been identified (38). Using the gene set enrichment analysis tool GINtool (39), we calculated the average fold changes of regulons, which reflects the regulator’s activity. Importantly, GINtool checks whether a regulator functions as an activator or as a repressor, which is important given that some regulators exhibit both activities. The results of the regulon analyses are listed in Table S2. Figure 3 presents a bubble matrix chart displaying the five most strongly activated and the five most strongly repressed regulons identified for each condition. The chart shows again that the xylose-inducible expression system exerts the most pronounced effect on host gene expression. Some regulons show a consistent response under all conditions, including the CssR regulon involved in secretion stress, the metabolic regulons AlsR, GlvR, and MelR, involved in acetoin synthesis, maltose, and melibiose utilization, respectively, and the YuxN regulon, involved in the regulation of Spx stability. The activity of the AraR, FadR, and HutP regulons, associated with arabinose utilization, fatty acid degradation, and histidine utilization, respectively, was primarily linked to PrgA overproduction. For the camelid nanobody and XynA, we could not detect a conserved regulon expression response. Finally, several metabolic regulons exhibit opposite activities in the two expression conditions, including AcoR, AhrC, CitR, G-box, GntR, PurR, PutR, PyrR, RocR, and TreR, which are involved in nucleoside, amino acid, and sugar metabolism. We have no obvious explanation for this.

### Improved PrgA production upon CtsR inactivation

**Fig 4:**
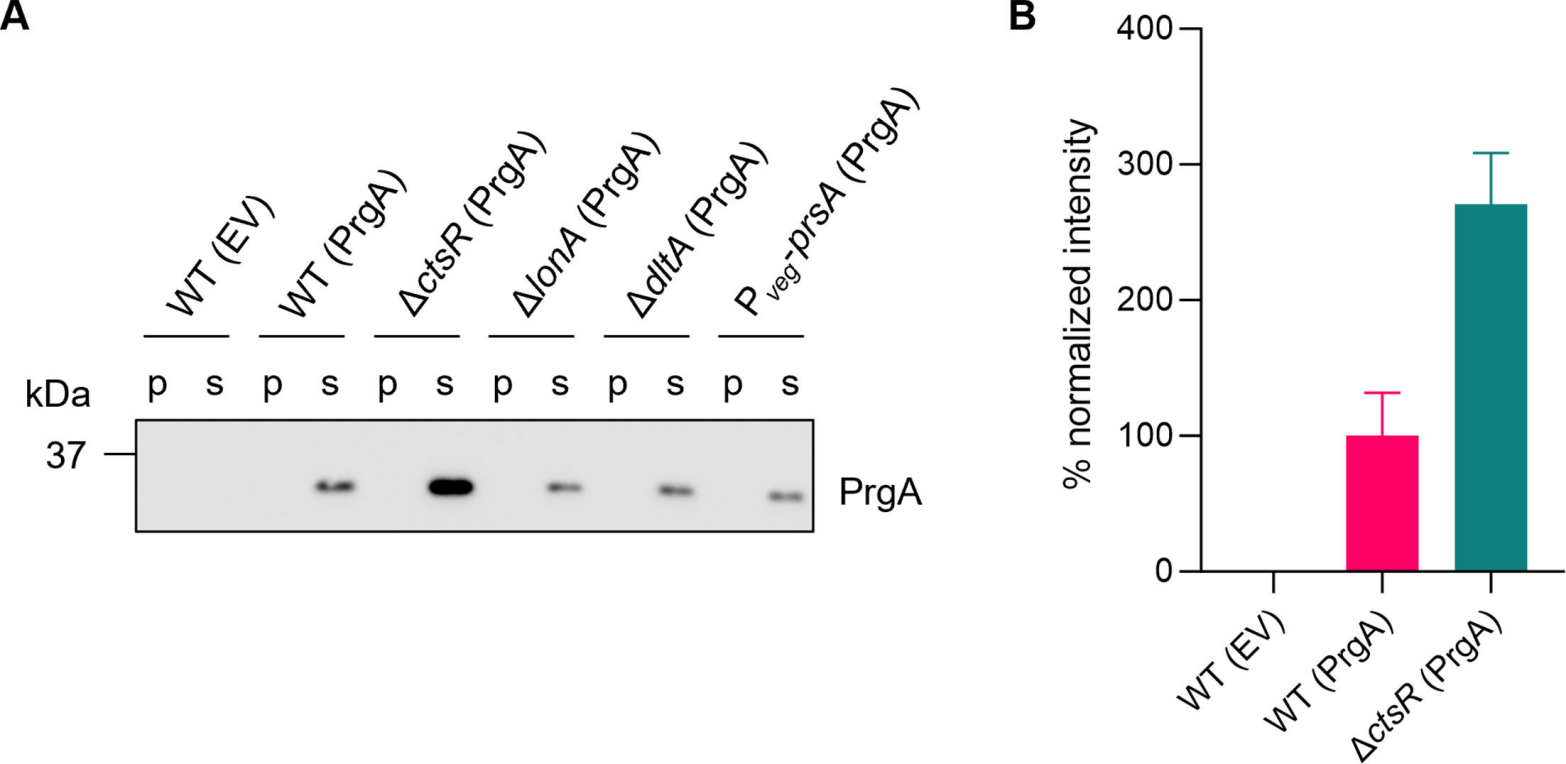
PrgA production in different *B. subtilis* mutants. (**A**) Western blot of PrgA levels in cell pellets (P) or the medium (supernatant, S). (**B**) Quantification of the supernatant western blot signals of three biological replicates of PrgA production and one empty vector control. Error bars reflect standard deviations.

Next, we tried to find information in the transcriptome data that could help to improve the yield of PrgA. Overexpression of PrgA strongly induces the expression of *htrA*, *htrB*, and *ykoJ*. This response mirrors findings reported in the context of α-amylase overproduction (8, 40, 41). The production of α-amylase has been successfully improved by (i) overproducing the extracellular lipoprotein PrsA, which is a protein folding chaperone, (ii) inactivation of the *dlt* operon (*dltX-dltA-dltB-dltC-dltD-dltE*), necessary for d-alanylation of teichoic acids, which increases the negative charge of the membrane, and (iii) deletion of the intracellular protease LonA (8, 42–46). Given the partial overlap in transcriptomic responses, it is plausible that similar genetic interventions could also improve PrgA production. To test this, we created three strains based on our background strain: two harboring deletions of *lonA* and *dltA*, and one strain carrying an extra copy of *prsA* under the control of the strong endogenous promoter P*veg*. However, when we introduced the constitutive PrgA expression plasmid into these engineered strains, we did not observe a significant increase in extracellular production (Fig. 4A).

In a previous transcriptome analysis focused on identifying production bottlenecks in XynA overexpressing *B. subtilis* strains, we observed a downregulation of several Clp protein chaperones (7). Inactivation of the transcriptional repressor CtsR, which controls expression of *clp* genes, resulted in a 25% increase in XynA production (7). Such mutation does not improve the production of the amylase amyM (8). Interestingly, *clpC* and *clpE* were also downregulated in cells constitutively overexpressing PrgA, albeit mildly, with log2 fold change of approximately 0.5–0.6, and therefore they are not present in either Table 1 or Fig. 3. Interestingly, when we evaluated the production of PrgA in a strain containing a *ctsR* deletion, we did observe a strong 2.7-fold increase in yield (Fig. 4). Of note, the observed PrgA protein bands correspond to its inactive, propeptide-containing form. Activation of PrgA requires proteolytic removal of this propeptide, which does not occur under the current experimental conditions, likely due to the short fermentation time and absence of the major secreted proteases AprE and NprE (17).

## DISCUSSION

This study was aimed at identifying general and protein-specific bottlenecks associated with the overproduction and secretion of three commercially relevant proteins by *B. subtilis*, including a camelid nanobody (GFPnb), a xylanase from *B. subtilis* (XynA), and the protein glutaminase PrgA from *C. proteolyticum*. To achieve this, we examined the transcriptome stress responses using RNA sequencing. In addition, we tested two different promoters, a constitutive and an inducible promoter. The largest gene expression differences were caused using the xylose-inducible P*xyl* promoter. In fact, xylose induction of GFPnb appeared to cause more transcription differences than the induction of PrgA, whereas the reverse is true for the constitutive overexpression conditions (compare Fig. 1 and 2). These effects may stem from the varying expression times, since the constitutive expression provides the host with time to adapt. Considering this, it will be interesting to evaluate transcriptome profiles when xylose is present continuously during growth. In any case, these results highlight the complexity of the cellular responses to protein overexpression.

 Since the efficiency of secretion depends on the secretion signal used, this domain will affect the transcriptome response. For GFPnb and PrgA, we used the heterologous secretion signal of YoaW (27). However, the transcriptome data from GFPnb and PrgA differed widely, making it impossible to deduce a transcriptome response that might be specific to the use of this signal sequence.

To contextualize our observations, we compared our transcriptome data to previously published data sets. Marciniak et al. analyzed the *B. subtilis* transcriptome responses related to a 30-min induction of *xynA*, using a subtilin-inducible promoter (6). Half of the differentially expressed genes that we identified when *xynA* was induced by xylose were also observed in their study (Fig. S3), including *htrAB, cssRS*, and genes of the *purE* operon. However, the transcriptome profile of constitutive XynA expression displayed almost no overlap with the transcriptome response described by Marciniak et al. (Fig. S3).

 As mentioned before, the transcriptome profiles of PrgA-producing strains showed stress responses that were reminiscent of what has been found for the overproduction of α-amylases (8, 40, 41). However, genetic interventions that have been shown to improve α-amylases production, including overexpression of the extracellular protein folding chaperone PrsA, changes in the cell wall teichoic acid composition by inactivating DltA or removal of the LonA protease, did not result in increased PrgA yields. Nevertheless, we were able to increase PrgA production 2.7-fold by inactivating the transcriptional regulator CtsR responsible for the transcriptional control of several Clp protein chaperones (47). This intervention ensued from a previous study where the XynA yield was improved by inactivating CtsR (7). A possible explanation for the downregulation of *clp* genes when cells are overexpressing a protein like XynA or Prg, is the fact that CtsR levels are controlled by the activity of ClpC and its partnerproteases ClpP (48). When ClpC is titrated by unfolded overexpressed proteins, it cannot inactivate its regulator CtsR, leading to higher levels of this repressor. Thi,s in turn, results in lower expression of *clp* genes, causing a self-enhancing effect (7). In the futur,e it will also be of interest to investigate whether oxidative folding of PrgA is a bottleneck, since its overexpression resulted in the upregulation of the thio-disulfide oxidoreductase *bdbB*. Of note, it has been shown before that upregulation of oxidoreductases can increase the yields of other heterologous proteins with disulfide bonds (49–51).

Based on our comprehensive transcriptome analyses, we must conclude that overexpression of a protein in *B. subtilis* does not necessarily lead to the up- or downregulation of unique genes, and that a common stress response cannot routinely be leveraged to make genetic changes that improve production, although the Δ*ctsR* mutation shows that it sometimes can. In addition, our transcriptome analyses demonstrate that, while *B. subtilis* host cells may produce the same protein, but the cellular responses are highly dependent on the expression conditions used. These variabilities pose challenges for leveraging transcriptomics, and potentially proteomics, as a tool to identify specific expression and secretion bottlenecks associated with particular proteins. It also underscores the importance of conducting such experiments under conditions that most closely mimic those of the industrial production process.

## MATERIALS AND METHODS

### Bacterial strains and growth conditions

*B. subtilis* strains used in this study are listed in Table S3. All expression hosts were derived from strain BWB143 (BSB1, trp+, Δ*aprE*, Δ*nprE*, and Δ*spoIIE*) (22). Cultures were grown in lysogeny broth (LB), composed of 1% (wt/vol) NaCl, 1% (wt/vol) tryptone, and 0.5% (wt/vol) yeast extract. When required, kanamycin (25–50 µg/mL) or spectinomycin (150 µg/mL) was supplemented to the medium. For overnight growth, the medium was supplemented with 0.5% (wt/vol) glucose. Induction of the P*xyl* protomer was achieved by adding xylose to a final concentration of 1% (wt/vol). Xylose was also added to the negative control containing an empty P*xyl* expression vector. Cultures were grown shaken at 210 rpm in a water bath of 37°C.

### Strain and plasmid construction

Naturally competent *B. subtilis* cells were used for constructing strains and plasmids (52). Strains BWB144, BWB145, and BWB146 were generated as previously described (7). Briefly, genomic DNA (gDNA) of the corresponding BKE library mutants (53) was transformed into competent BWB143 cells, and the resulting gene deletions were verified by PCR. The *prsA* overexpression strain BKM02 was created by introducing an *amyE::Pveg-prsA, spec* construct into BWB143. This construct was assembled using overlap extension PCR with three fragments: (i) the upstream *amyE* sequence, (ii) the *prsA* sequence, and (iii) the *spec* marker with the downstream *amyE* sequences. Primer sequences are provided in Table S4. gDNA from strain BCS376, which contains the *amyE::xynA, spec* construct (lab storage), served as the template for fragments 1 and 3. Fragment 1 was amplified using primers MK049 and MK050, and fragment 3 was amplified using primers BW_433 and GD-161. BSB1 gDNA was used as the template for fragment 2, amplified using primers MK047 and MK048. Primers MK047 and MK050 contain the sequence of P*veg* from the *B. subtilis* 168 genome. The three fragments were combined by overlap PCR using primers MK049 and GD-161. The final product was verified by sequencing and subsequently transformed into competent BWB143 cells. Transformants were selected on spectinomycin-containing LB agar plates and validated by PCR.

A complete list of plasmids used in this study is available in Table S5. The proteins of interest are fused with a 6x-histidine tag to facilitate detection by western blotting. Plasmids pMKC01 (P*amyQ*-YoaWss-GFPnb-His6) and pMKC02 (P*amyQ*-YoaWss-PrgA-His6) were constructed via Gibson assembly. This involved combining the pCS58 backbone, linearized with PacI and HindIII-HF, with gene inserts amplified by PCR using primers MK029 and MK030 (Table S4), and plasmids pMKX04 and pMKX05 as template. All enzymes and reagents used in these procedures were obtained from New England Biolabs (NEB). The assembled constructs were transformed into *B. subtilis* and validated by sequencing.

### RNA sequencing

The RNA sequencing protocol was based on references 54, 55. Bacterial strains containing the relevant plasmids were cultured overnight and then diluted to an OD_600_ equivalent of 0.05 in fresh LB medium. Cultures were incubated at 37°C with shaking at 210 rpm in a water bath. Expression from the xylose-inducible plasmids was induced by adding xylose to a final concentration of 1% after 3 h, corresponding to the end of the logarithmic growth phase. Samples were collected 2 h after induction for subsequent analysis by SDS-PAGE (see below) and RNA isolation. For cells constitutively expressing the constructs, samples were taken 5 h after the initial dilution. For RNA isolation, 450 µL of bacterial culture was transferred into 2 mL screw-cap Eppendorf tubes containing 1.5 g of 0.1 mm glass beads, 500 µL phenol:chloroform:isoamyl alcohol (PCI; 25:24:1, Carl Roth), 50 µL 10% SDS, and 50 µL RNase-free water. The samples were vortexed, flash-frozen in liquid nitrogen, and stored at −80°C until further processing. Cells were lysed using a bead beater (Precellys 24) for eight cycles at 6,000 rpm, each lasting 25 s with 2 min cooling intervals on ice in between cycles. The lysed samples were then centrifuged for 5 min at 15,871 RCF (full speed table top centrifuge) at 4°C. The upper aqueous phase (400 µL) was transferred to a new tube and mixed with 400 µL chloroform, vortexed, and centrifuged for 2 min at 21,130 RCF at 4°C. From the resulting aqueous phase, 300 µL was transferred to a new tube and mixed with 700 µL ice-cold EtOH/3 M NaAc (pH 5.2, 30:1 ratio), mixed by inversion and incubated at −80°C for 1 h. After incubation, the samples were centrifuged for 30 min at full speed at 4°C. The supernatant was discarded and the RNA pellet was washed with ice-cold 70% EtOH. Following a 5-min centrifugation at the same speed and temperature, the pellet was air-dried for 5–10 min at RT. The dried pellet was resuspended in 100 µL RNase-free water and incubated at 65°C with shaking at 1,000 rpm for 5 min in a tabletop shaker. A 50 µL aliquot was stored at −80°C as a backup, while the remaining 50 µL was treated with 1 µL DNase (Thermo) and incubated at 37°C for 1 h to remove residual DNA. After DNase treatment, 240 µL RNase-free water and 300 µL PCI were added to the sample. The mixture was vortexed and centrifuged for 10 min at full speed at 4°C. From the upper aqueous phase, 250 µL was transferred to a new tube and combined with 500 µL EtOH/3 M NaAC (pH 5.2, 30:1 ratio), mixed by inversion and incubated at −80°C for 1 h. The RNA was then pelleted by centrifugation for 30 min at full speed at 4°C. The supernatant was discarded, and the pellet was washed with ice-cold 70% ethanol, centrifuged for 5 min at the same speed, and air-dried. Finally, the pellet was dissolved in 20 µL RNase-free water. The RNA concentration was measured using a NanoDrop spectrophotometer, the quality of the samples was inspected on a 1% TBE-agarose gel, and samples were stored at −80°C for further use.

rRNA was depleted using complementary primers and RNase H digestion (56). For each RNA sample, 2 µg was diluted to a final volume of 10 µL in a PCR tube. A custom rRNA oligo mix (2 µL) and 4 µL probe hybridization buffer (0.8 M NaCl, 0.4 M Tris-HCl pH 7.5) were added. The mixture was incubated in a thermocycler using the following program: 95°C for 2 min, gradual cooling to 45°C at a rate of 0.1°C/s, holding at 45°C for 5 min. After hybridization, 2 µL RNase H reaction buffer (NEB), 1 µL NEBNext Thermostable RNase H (NEB), and 1 µL nuclease-free water were added. The sample was incubated in the thermocycler at 50°C for 90 min with the lid temperature set to 55°C. Next, 5 µL DNase I reaction buffer, 2.5 µL NEBNext DNase I (RNase-free), and 22.5 µL nuclease-free water were added to the reaction. The sample was incubated in a thermocycler at 37°C for 60 min with the lid temperature set to 40°C. The reaction (50 µL) was transferred to a 2 mL Eppendorf tube and diluted with 200 µL nuclease-free water. Then, 250 µL PCI was added, and the mixture was centrifuged for 10 min at full speed at 4°C. From the upper aqueous phase, 200 µL was transferred to a new tube. To precipitate the RNA, 400 µL of EtOH/3M NaAc (pH 5.2, 30:1) and 1 µL of Glycoblue (Thermo Fisher) were added. The solution was mixed and incubated at −80°C for 1 h. The RNA was pelleted by centrifugation for 30 min at full speed at 4°C. The pellet was washed two times with 70% ice-cold EtOH, air-dried for 5 min at RT, and resuspended in 9 µL nuclease-free water. The RNA concentration was measured using the Qubit RNA HS kit following the manufacturer’s protocol.

RNA sequencing libraries were prepared using the NEBNext Ultra II Directional RNA Library Prep Kit for Illumina in combination with NEBNext multiplex oligos for Illumina, following the manufacturer’s protocol. Of note, one-quarter of the recommended RNA input and reagent volumes was used during the preparation. After amplification of the library, the quality and quantity of the samples were inspected by 8% TBE 7 M Urea PAGE. For purification steps, AMPure XP beads (Beckman Coulter) were used instead of NEBNext sample purification beads. RNA sequencing was performed on an Illumina NextSeq 550 sequencing system using the NextSeq 500/550 High Output v2.5, producing 75 bp reads. The sequencing data were processed using the web-based platform Galaxy (usegalaxy.org). Read quality was assessed with FastQC (57), and trimming was performed using Trimmomatic (58). The trimmed reads were aligned to the reference genome by Bowtie2 (59, 60), and gene feature counts were obtained using featureCounts (61). Differential gene expression analysis was conducted with DESeq2 by comparing transcriptomes of overexpression strains to those of the empty vector control (62).

Regulon activity analyses were carried out with GINtool (version 1.0.0.73) (39). To determine the most probable average activity levels for each regulon, the “Best score” column from the “Mapping_details” sheet of the GINtool output was used. Reference files, including “example gene info file,” “example regulons file,” and “example regulon info file,” were sourced from the GINtool starter package and were originally derived from the *B. subtilis* online database “SubtiWiki” (38). Visualization of growth curves, volcano plots, and dot plots was performed using GraphPad Prism, while bubble matrices were generated using a custom R script.

### SDS-PAGE

Pellet and supernatant samples for SDS-PAGE were prepared by centrifuging 1 mL of bacterial culture at 21,300 RCF for 1 min. For the supernatant, 700 µL was transferred to a new tube containing 700 µL 20% trichloroacetate (TCA) in 50% acetone, while the remaining supernatant was discarded. The TCA-supernatant mixture was incubated on ice for at least 5 min before centrifugation for 20 min at 21,300 RCF at 4°C. The supernatant was removed, and the pellet was washed with 500 µL ice-cold acetone, followed by another centrifugation under the same conditions. After the acetone supernatant was discarded, the pellets were air-dried for 5 min and dissolved in 2× sample buffer (0.125 M Tris pH 6.8, 4% SDS, 20% glycerol, 0.02% bromo phenol blue, and 20 µM DTT) to an equivalent of 100 OD_600_ units. The dissolved samples were then mixed 1:1 with PBS. For the pellet samples, the bacterial pellets were resuspended in PBS containing 0.25 µg/mL lysozyme, 1× cOmplete protease inhibitor cocktail (Roche), and 1 mM PMSF to achieve a concentration equivalent to 10 OD_600_ units. The samples were incubated in a tabletop shaker at 1,000 rpm and 37°C for 15 min, after which they were diluted 1:1 in 2× sample buffer. Both pellet and supernatant samples were heated at 98°C for 10 min immediately before loading onto the SDS-PAGE gel. Following electrophoresis, the gel was incubated in fixative solution (50% methanol and 10% acetic acid) for 20 min, stained with Coomassie Brilliant Blue (0.05% G250 CBB and 10% acetic acid) for 30 min and washed with destain solution (10% acetic acid) for 1 h until the background of the gel became clear. Finally, the gel was scanned using a Bio-Rad GS-800 scanner to document the results.

### Western blotting

Pellet and supernatant samples were separated by 12% SDS-PAGE, with pellet samples loaded at an equivalent of 0.015 OD units and supernatant samples at an equivalent of 0.15 OD units. Following electrophoresis, proteins were transferred to a nitrocellulose membrane and incubated overnight at 4°C in blocking buffer (1% [wt/vol] skim milk powder in TBS-Tween20). The membrane was then incubated with an α-His6 antibody (1:500, Mouse, Roche, catalog number 11922416001) for 1 h. After three 3-min wash steps, the membrane was incubated with an HRP-tagged α-Mouse antibody (1:5,000, Goat, Bio-Rad) for 1 h. Following a further three wash steps with blocking buffer, the membrane was incubated for 1 minwith Lumi-Light western blotting substrate (Roche). The chemiluminescent signal was detected using an AI600 imager for 5 s, and the signal intensities were quantified using Image Lab Software (Bio-Rad).

## Data Availability

Transcriptome data are available in the NCBI Gene Expression Omnibus under accession number GSE293838 (63).

## SUPPLEMENTAL MATERIAL

Supplemental material aem.01374-25-s0001.pdf Tables S3 to S5; Fig. S1 to S3.

Table S1 aem.01374-25-s0002.xlsx Differential expression analysis.

Table S2 aem.01374-25-s0003.xlsx GINtool regulon activity analysis.

ASM does not own the copyrights to Supplemental Material that may be linked to, or accessed through, an article. The authors have granted ASM a non-exclusive, world-wide license to publish the Supplemental Material files. Please contact the corresponding author directly for reuse.
