# Supplementary material for: Impact of expression systems on the transcriptome of Bacillus subtilis: insights for enhanced production of glutaminase PrgA
Source: Appl Environ Microbiol. 2025 Sep 4;91(10):e01374-25. doi: 10.1128/aem.01374-25 (PMC12542772; doi:10.1128/aem.01374-25)
Supplement: Supplemental material — Tables S3 to S5; Fig. S1 to S3. [file aem.01374-25-s0001.pdf]

Impact of Expression Systems on the Transcriptome of *Bacillus subtilis*: Insights for Enhanced Production of Glutaminase PrgA

Mariah Kes, Biwen Wang, Joen Luirink, Leendert Hamoen

Content:

Table S3: *Bacillus subtilis* expression hosts used in this study

Table S4: Primers used in this study

Table S5: Plasmids used in this study

Figure S1: Growth curves of overexpressing strains

Figure S2: Secreted protein levels

Figure S3: Comparison of *xynA* overexpression transcriptome datasets

Table S3: *Bacillus subtilis* expression hosts used in this study.

Strain	Genotype	Source
BWB143	BSB1 trp+ Δ*aprE*, Δ*nprE*, Δ*spoIIE*	(1)
BWB144	BSB1 trp+ Δ*aprE*, Δ*nprE*, Δ*spoIIE*, Δ*ctsR::ery*	This work
BWB145	BSB1 trp+ Δ*aprE*, Δ*nprE*, Δ*spoIIE, ΔlonA::ery*	(2)
BWB146	BSB1 trp+ Δ*aprE*, Δ*nprE*, Δ*spoIIE, ΔdltA::ery*	This work
BKM02	BSB1 trp+ Δ*aprE*, Δ*nprE*, Δ*spoIIE, amyE::Pveg-prsA, spec*	This work

Table S4: Primers used in this study.

Name	Sequence
GD-161	ATGGGGAAGAGAACCGCTTA
BW_433	TAGTTTCGAAAAAAGGCCGC
MK029	CTTTACCTTGTCTCCAAGCTTCTTCTAGAGGTACCGCATGCCTCCAGCAATTCCAAGGCC
MK030	GAGAGGGAGAGGAAATTAATTAAAAAAGGAGCGATTTACATATGAAAAAGATGTTGATGTTAGCT
MK047	TAATTTAAATTTTATTTGACAAAAATGGGCTCGTGTTGTACAATAAATGTAGTGAGGTGGATGCAATG AAGAAAATCGCAATAGCAG
MK048	GGGCGGCCTTTTTTCGAAACTATTATTTAGAATTGCTTGAAGATGAA
MK049	ATGTTTGCAAAACGATTCAAAAC
MK050	AGCCCATTTTTGTCAAATAAAATTTAAATTAGCGGCCGCGATTTCTC

Table S5: Plasmids used in this study.

Plasmid	Promotor	Protein construct	Source
pCS74	P*amyQ*	Empty vector (EV)	(3)
pCS58	P*amyQ*	XynAss-XynA	(4)
pMKC01	P*amyQ*	YoaWss-GFPnb-His6	This study
pMKC02	P*amyQ*	YoaWss-PrgA-His6	This study
pMKX01	P*xyl*	Empty vector (EV)	(1)
pMKX03	P*xyl*	XynAss-XynA	(1)
pMKX04	P*xyl*	YoaWss-GFPnb-His6	(1)
pMKX05	P*xyl*	YoaWss-PrgA-His6	(1)

Figure S1

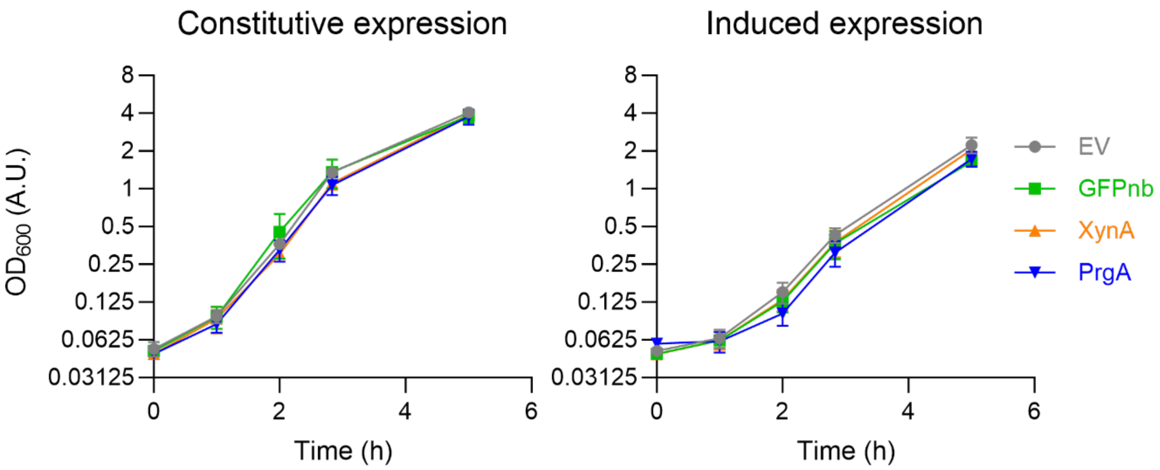

Figure S1: Growth curves of overexpressing strains. Strains expressing GFPnb, XynA, PrgA, or harboring the empty vector were grown for 5 h. Expression was induced at t = 3 h. Error bars reflect standard deviations of 2 biological replicates (except for C-EV and C-XynA at t = 2 h, 1 replicate is shown).

Figure S2

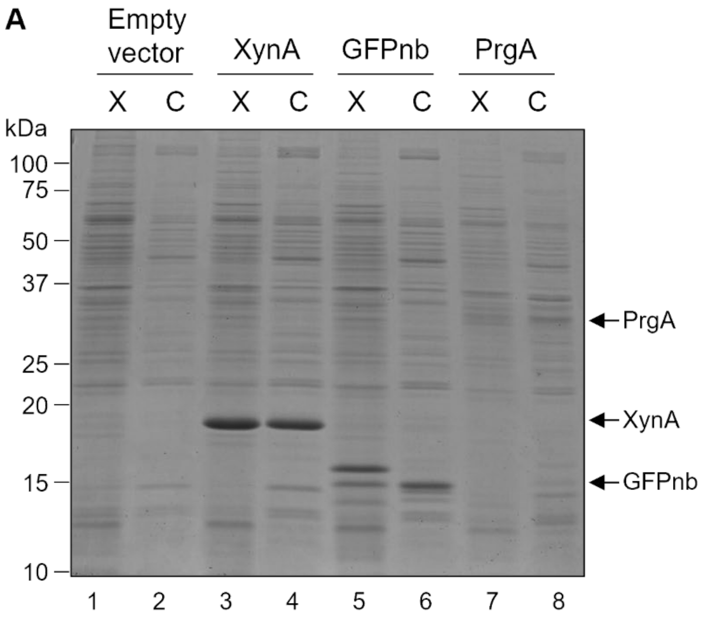

Figure S2: Secreted protein levels. A) Coomassie Brilliant Blue (CBB)-stained SDS-PAGE of supernatant protein samples at RNA sampling timepoint. Arrows indicate expected heights of PrgA, XynA and GFPnb (from top to bottom). X: xylose-inducible expression; C: constitutive expression. **B**) CBB-stained SDS-PAGE of supernatant protein samples of time series after induction of GFPnb expression.

Figure S3

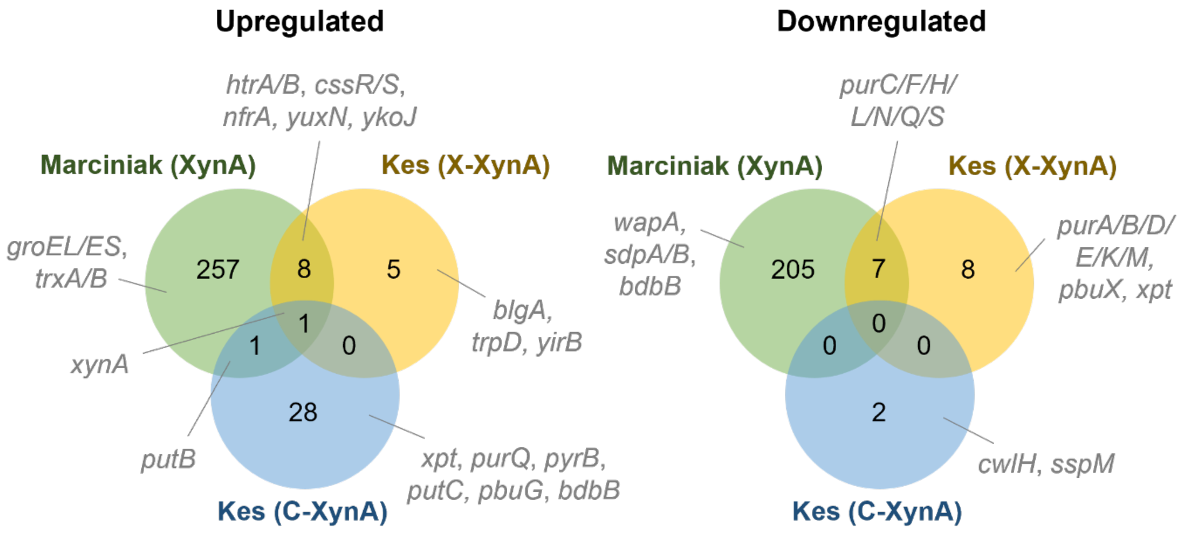

Figure S3: Comparison of *xynA* overexpression transcriptome datasets. The *xynA* overexpression transcriptome dataset of Marciniak *et al.* (2012) (5) was compared to our datasets (Kes) of induced (X-XynA) and constitutive (C-XynA) *xynA* overexpression. We show the significant genes with a FC > 2 for Marciniak (XynA) and Kes (X-XynA) and FC > 1.5 for Kes (C-XynA) and a p-value < 0.05 for all datasets.

References

1. Kes MBMJ, Wang B, van Ulsen P, Hamoen LW, Luirink J. 2024. Development of a split-luciferase assay to establish optimal protein secretion conditions for protein production by Bacillus subtilis. Microbiology (Reading) 170.

2. Wang B, Kes MBMJ, van Saparoea ACH van den B, Dugar G, Luirink J, Hamoen LW. 2024. Inactivation of the conserved protease LonA increases production of xylanase and amylase in Bacillus subtilis. Microb Cell Fact 23.

3. Henriques G, McGovern S, Neef J, Antelo-Varela M, Götz F, Otto A, Becher D, van Dijl JM, Jules M, Delumeau O. 2020. SppI Forms a Membrane Protein Complex with SppA and Inhibits Its Protease Activity in Bacillus subtilis. mSphere 5.

4. Wang B, van der Kloet F, Hamoen LW. 2023. Induction of the CtsR regulon improves Xylanase production in Bacillus subtilis. Microb Cell Fact 22.

5. Marciniak BC, Trip H, van-der Veek PJ, Kuipers OP. 2012. Comparative transcriptional analysis of Bacillus subtilis cells overproducing either secreted proteins, lipoproteins or membrane proteins. Microb Cell Fact 11.
